# A haplotype of three SNPs in *FTO* had a strong association with body composition and BMI in Iranian male adolescents

**DOI:** 10.1371/journal.pone.0195589

**Published:** 2018-04-20

**Authors:** Naser Kalantari, Nastaran Keshavarz Mohammadi, Pantea Izadi, Saeid Doaei, Maryam Gholamalizadeh, Hassan Eini-Zinab, Tuire Salonurmi, Shahram Rafieifar, Reza Janipoor, Ghasem Azizi Tabesh

**Affiliations:** 1 Department of community nutrition, School of Nutrition and Food Sciences, Shahid Beheshti University of Medical Sciences, Tehran, Iran; 2 Department of public health, Shahid Beheshti University of Medical Sciences, Tehran, Iran; 3 Department of Human Genetics, Department of human genetics, School of Medicine, Tehran University of Medical Sciences, Tehran, Iran; 4 Natural Products and Medicinal Plants Research Center, North Khorasan University of Medical Sciences, Bojnurd, Iran; 5 Student Research Committee, Cancer Research Center, Shahid Beheshti University of Medical Sciences, Tehran, Iran; 6 Department of public health, School of Public Health, North Khorasan University of Medical Sciences, Bojnurd, Iran; 7 Research groups of Internal Medicine, Faculty of Medicine, University of Oulu, Oulu, Finland; 8 Health Promotion and Education Department, Ministry of health & medical education, Tehran, Iran; 9 Department of Veterinary Medicine, School of Veterinary Medicine, Shiraz University of medical sciences, Shiraz, Iran; 10 Department of Human Genetics, Faculty of Medicine, Shahid Beheshti University of Medical Sciences, Tehran, Iran; Universidade de Sao Paulo, BRAZIL

## Abstract

**Background:**

Single-nucleotide polymorphisms (SNPs), which are located in the first intron of the *FTO* gene, are reported to be associated with body weight and the body mass index (BMI). However, their effects on anthropometric measurements in adolescents are poorly understood.

**Objective:**

This study aimed to investigate the association of three adjacent polymorphisms (rs9930506, rs9930501, & rs9932754) in the *FTO* gene with anthropometric indices in Iranian adolescent males.

**Design:**

The participants comprised a total of 237 adolescent males who were recruited randomly from two high schools in Tehran, Iran. The DNA samples were genotyped for the *FTO* gene polymorphisms by DNA sequencing. BMI, body fat percentage (BF%), and body muscle percentage (BM%) were determined using a validated bioelectrical impedance analysis scale. The association of the *FTO* polymorphisms with weight, height, BMI, BF%, and BM% was investigated.

**Results:**

A haplotype of rs9930506, rs9930501, and rs9932754 (GGT) in the first intron of the *FTO* with complete linkage disequilibrium (LD) was found to be significantly associated with higher weight (OR = 1.32), BMI (OR = 5.36) and BF% (OR = 1.46), and lower BM% (OR = 3.59) (all P<0.001). None of the students with GGC genotypes were underweight, while all of the students with AAT genotypes had high muscle mass.

**Conclusions:**

A haplotype in the first intron of the *FTO* gene had a strong association with obesity indices in Iranian adolescent males. The *FTO* gene polymorphisms might have greater effects on anthropometric indices than what was previously imagined. Moreover, we suggested that the *FTO* gene exerted their effects on anthropometric measurements through haplotypes (and not single SNPs).

## Introduction

A genome-wide association study (GWAS) has identified 835 genes and 317 gene polymorphisms that were related to obesity [1]. The role of polymorphisms of several genes (e.g. *FTO* [2], *PRAR* [3], *adrenergic beta receptor* [4], and *Perilipin* [5] in obesity has been frequently discussed. Among these genes, the *FTO* gene is reported as the most important gene factor related to obesity [6–8].

The *FTO* gene is located on chromosome 16 and encodes a 2-oxoglutarate-dependent nucleic acid demethylase that catalyzes the demethylation of 3-methylthymine in single-stranded DNA [9]. A duplication of the *FTO* gene leads to mental retardation, obesity, and some other abnormalities [10]. The *FTO* gene is expressed in many tissues, although the highest expression level of this gene is expressed in brain and hypothalamus [11]. Several studies have reported the relationship between *FTO* and obesity [12–15]. However, there is no general consensus on the underlying mechanisms of the effects of *FTO* on body weight. Recent studies showed that polymorphisms of *FTO* play a key role in the control of food intake. People with the AA or AT genotypes of rs9939609 polymorphism had a significantly higher calorie intake than people with the TT genotype [12]. Moreover, the obesity risk allele of *FTO* was associated with a lower level of lipolysis in adipocytes, which shows the possible primary role of the *FTO* gene in adipose tissue [13]. Many polymorphisms of the *FTO* gene were studied for the possible association with obesity. Some of these SNPs are rs178117449, rs9939609, rs3751812, rs1421085, rs9930506, and rs7202116 [14]. Recent studies have also shown that the genotype of the rs9930506 polymorphism has the strongest influence on body weight and body composition [14,15].

However, the relationship of the rs9930506 polymorphism with anthropometric measurements in adolescents is not clear. Hence, this study used DNA sequencing to assess the genotype of the rs9930506 polymorphism and two other adjacent polymorphisms (rs9930501 & rs9932754) in the first intron of the *FTO* gene. The association between these SNPs and anthropometric indices (i.e. weight, height, BMI, BF%, and BM%), and the possible correlation between SNPs in Iranian adolescent males were investigated.

## Materials and methods

### Study population

This paper reports the first phase of a comprehensive interventional study (interactions of genetics, lifestyle and anthropometrics study, or IGLA study) and is registered in the Iranian Registry of Clinical Trials as IRCT2016020925699N2. The first phase of this study was designed as a cross-sectional study on 280 adolescent male students of two schools from one district of Tehran over one school year from January to June 2016 (140 students of each school). Based on the agreement of the schools’ authorities, and on the similarity in terms of socio-demographic background and the rate of obesity among school students in one district in Tehran, two high schools were selected to participate in the study. The inclusion criteria were the students’ age (12–16 years), their willingness to participate in the study, and the puberty stage. The stage of puberty was assessed by a pediatric psychologist using the Tanner criteria of genital and pubic hair stage. Students suffering from diseases that affect body weight (n = 20), students who were treated with drugs that impact body weight (n = 14), and students who were afraid of blood sampling (n = 5) and had difficulty finding the veins (n = 4) were excluded. Finally, a total of 237 adolescent males were studied (125 students of one school and 112 students of another school).

### Anthropometric assessments

All students were assessed in terms of anthropometric parameters (i.e. weight, height, BMI, body fat percentage (BF%), and body muscle percentage (BM%)). Heights were measured in the standing position and without shoes using a standard measuring tape attached to the wall. Students’ weights were measured, and the values of BMI, BF and BM were determined using a validated bioelectrical impedance analysis scale (Omron BF-511) [16] after entering their age, gender, and height. The extracted data was classified according to World Health Organization z-scores (for height, weight, and BMI) [17] and the published criteria reported by recent studies (for BF and BM) [18]. Moreover, the maturity situation of the students was examined by a psychologist.

### Genotyping

The FTO gene encompasses >410 kb of genomic region. Therefore, a targeted approach was adopted to identify genetic variations located within 300 base pairs (bps) upstream and downstream of the SNP rs9930506, which is strongly associated with obesity [19]. Five ml of blood samples were collected in EDTA tubes from each participant. The samples were then transported to the cellular and molecular laboratory at the school of nutritional sciences and food technology of Shahid Beheshti University of Medical Sciences for buffy coat isolation, DNA extraction, and PCR.

The DNA extraction kit manufactured by GeneAll was used to extract and purify the DNA samples. The NanoDrop device (Thermo Scientific, Wilmington, DE, USA) was used to quantify the DNA concentration. The optical density (OD) of the samples was obtained in the absorption rate of 260–280, and it was confirmed if the OD was from 1.8 to 2. Moreover, to check the quality of the extracted DNA, electrophoresis using the agarose gel technique was used. In brief, genomic DNA was amplified by PCR using the Taq DNA Pol 2X Master Mix Red (cat. No A180301; Ampliqon, Denmark). The PCR products were sent to GeneAll for DNA sequencing. The quality and average length of the sequence library for each sample was assessed using the Chromas software (version 2.33, http://www.technelysium.com.au/chromas.html).

### Statistical analyses

Statistical analyses were performed using the SPPS software (version 23). The population was tested to determine whether the genotypes were in Hardy–Weinberg equilibrium by comparing the observed genotype frequencies in AAA cases and controls with their expected frequencies. The association between SNPs and quantitative anthropometric measures was evaluated by linear regression and logistic regression after adjusting for age. P-values of <0.05 were considered significant for all statistical tests.

### Ethics statement

The study was conducted at the Department of Community Nutrition of the Shahid Beheshti University of Medical Sciences (Tehran, Iran), and was approved by the ethics committee of the National Nutrition and Food Technology Research Institute, Tehran, Iran (reference number: Ir.sbmu.nnftri.rec.1394.22). The details of the study were explained to the students and their parents along with an explanatory letter, and written informed consent was obtained from the parents and the students prior to their joining the project.

## Results

The students’ age and weight mean were about 14 years and 61 kg respectively (Table 1), and all of the students had passed the sexual maturity stage. More than half the students had normal BMI and above-average BM. More than 40% of them were overweight or obese, and 22.8% had above-average FM.

**Table 1:** Characteristics of the participants (n = 237).

Demographic indices	Mean	SD
Students’ age (years)	14.1	1.27
Students’ height (cm)	169.33	9.84
Students’ weight (kg)	61.45	16.37
Students’ BMI (kg/m^2^)	22.05	4.77

### The *FTO* genotype

A complete LD in a haplotype block composed of three SNPs (rs993275 (allele G), rs9930506 (allele G), rs9930501 (allele C)) encompassing 40 bps in all the students was identified. Thus, we reported only rs9930506 for the following analyses. The A and G allele frequencies for rs9930506 polymorphism were 0.6 and 0.4, respectively, and the genotypes were in the Hardy–Weinberg equilibrium (number of observed AA, AG, and GG genotypes = 83, 116, and 38, respectively; number of expected CC, CT, and TT genotypes = 84, 114, and 39, respectively; P = 0.761). About half the students had the heterozygous genotype, 35% had the homozygous genotype AA, and 16% had the homozygous genotype GG. The average length of sequence was 420 bps. A summary of the anthropometric measurements according to the three genotypes are presented in Table 2.

**Table 2:** Summary statistics of anthropometric measurements (N (%)).

Anthropometric indices		AA (n = 85)	AG (n = 116)	GG (n = 38)	Total (n = 237)
BMI	Underweight (under –2 Z-score)	5 (2.11)	4 (1.69)	0 (0)	9 (3.8)
	Normal	49 (20.67)	73 (30.8)	8 (3.37)	130 (54.85)
	Overweight (higher than +1 Z-score)	18 (7.59)	19 (8.02)	10 (4.22)	47 (19.83)
	Obese (higher than +2 Z-score)	11 (4.64)	20 (8.44)	20 (8.44)	51 (21.52)
BF%	Low (under percentile 2)	22 (9.28)	25 (10.55)	2 (0.84)	49 (20.67)
	Normal	49 (20.67)	73 (30.8)	18 (7.59)	140 (59.07)
	High (higher percentile 91)	5 (2.11)	8 (3.37)	7 (2.95)	20 (8.44)
	Very High (higher percentile 98)	7 (2.95)	10 (4.22)	11 (4.64)	28 (11.82)
BM%	Low (under percentile 2)	0 (0)	1 (0.42)	1 (0.42)	2 (0.85)
	Normal	32 (13.5)	53 (22.36)	31 (13.08)	116 (48.94)
	High (higher percentile 98)	51 (21.52)	62 (26.16)	6 (2.53)	119 (50.21)

BMI: Body mass index, BF: Body fat, BM: Body muscle.

### Association between the rs9930506 polymorphism and anthropometric indices

**Fig 1:**
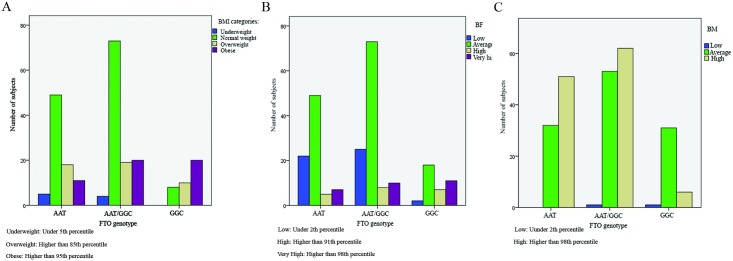
Anthropometric distribution of subjects with different *FTO* genotypes. (A) All of the students with the GGC genotype of these polymorphisms had normal or higher BMI. (B) People with the GGC genotype of these polymorphisms had significantly higher BF (C). All of the students with the AAT genotype of these polymorphisms had normal or higher BM. BMI: Body mass index, BF: Body fat%, BM: Body muscle%.

Our results showed that people with the GG genotype of this polymorphism had significantly higher weight (OR = 2.90), BMI (OR = 5.36) and BF (OR = 1.46), and lower BM (OR = 3.59) than subjects with the AA and AT genotypes (all P<0.001) (Table 3). As shown in Fig 1, all of the students with the GGC genotype of these polymorphisms had normal or higher weight and BMI. Besides, those with the AAT genotype had higher BM than other students, and none of them was in the low BM group (Fig 1).

**Table 3:** The relationship between the GG genotype of rs9930506 polymorphism with anthropometric indices as dependent variables.

anthropometric indices	Beta coefficient	P value	OR	CI
weight (kg)	0.22	0.001	2.90	.93–9.06
height (cm)	-0.09	NS	1.32	.98–1.8
BMI (kg/m^2^)	0.27	0.001	5.36	2.39–11.99
BF (%)	0.3	0.001	1.46	1.08–1.97
BM (%)	-0.28	0.001	3.59	1.61–8.01

Beta coefficient was determined using linear regression and OR (odds ratio) was determined using logistic regression after adjusting for age (n = 237). BMI, body mass index; BF, body fat, BM, body muscle; NS, not significant.

## Discussion

The results of the study showed that none of the students with GGC genotype were underweight while most of the students with AAT genotype had high BM. The minor allele frequency (MAF) was 40%, which was close to the findings in white populations [15]. However, the frequency of these alleles is different in non-white populations. For example, Tong et al. reported 17% MAF in a Chinese population, which was much less than that in the present study [20].

The relationship between rs9930506 polymorphism and anthropometric measurements has been studied frequently. The GG genotype of the rs9930506 polymorphism was positively correlated with weight, BMI, and BF, while it was negatively correlated with the BM. For example, in a study by Scuteri et al. to evaluate the polymorphisms associated with obesity, the results showed that the rs9930506 polymorphism had the strongest correlation with BMI [21]. Also, in a study conducted by Sentinelli et al. to determine the effects of rs9930506 and rs9939609 polymorphisms on BMI, the results showed that both polymorphisms were associated with higher BMI, and that the rs9930506 polymorphism showed a stronger association. In addition, both SNPs were correlated with the early onset of childhood obesity [15]. In another study by Coto et al. carried out among an elderly Caucasian–Spanish population, the frequency of the GG genotype of the rs9930506 polymorphism in people with a BMI higher than 25 was significantly higher than other subjects [22]. However, some studies have reported contradictory results. Li et al. investigated the association between three polymorphisms of the *FTO* gene (rs8050136, rs9939609, and rs9930506) with obesity, and the results showed that none of the studied SNPs were associated with obesity and waist circumference [20]. Moreover, Wang et al. studied the relationship between *FTO* polymorphisms (including rs9930506) and metabolic syndrome, and the rs9930506 polymorphism did not show a significant relationship with the pathogenesis of metabolic syndrome including abdominal obesity [23]. However, both these negative studies were conducted on Chinese people. As mentioned above, the MAF of this polymorphism in Chinese communities is significantly less than that in European populations [21].

Two other SNPs (rs9930501 & rs9932754) have received less research attention with respect to their potential impact on anthropometric measurements [24–25]. In a study on common variations in the *FTO* gene among Thai people, no association was found between rs9930501 polymorphism and obesity [24]. In another study, the association between rs9932754 and BMI and its same MAF with rs9930506 was reported [25].

The mechanisms beyond the effect of the *FTO* gene polymorphisms on anthropometric measures are not clear. It has been reported that these SNPs may influence the *FTO* gene expression level [26]. Moreover, the effects of dietary components on the *FTO* gene expression have been reported recently [27]. It is possible that the *FTO* gene polymorphism can modify the effects of diet on the *FTO* gene expression [28]. Although recent studies suggested that *FTO* has an effect on BMI, at least partly, through the change in the expression levels of other obesity-related genes such as *IRX3* [29–30], Moreover, a complete LD was found in the present study between three neighboring SNPs of the first intron region of the *FTO* gene. Although the linkage disequilibrium (LD) between rs9930506, rs9930501 and rs9932754 has been reported in genome-wide association studies [22, 31, and 32]. The results of the present study suggest that the *FTO* gene polymorphisms play their roles as a haplotype (and not single SNPs). Given the close proximity of these polymorphisms, a new hypothesis could be that these polymorphisms exert their effects on gene expression as a sequence of the first intron region. However, there are some limitations of the study that need to be considered: The findings of this study are limited to adolescent males and the sample size is relatively small. A further study with a larger sample size is needed to confirm our results.

## Conclusion

The results of this study showed that a haplotype in the first intron of the *FTO* gene had a strong association with obesity indices among Iranian adolescent males. None of the students with GGC genotypes were underweight, while most of the students with AAT had high BM. Thus, it can be concluded that the *FTO* gene polymorphisms might have greater effects on anthropometric indices than what was previously imagined. Moreover, we suggested that the *FTO* SNPs exert their effects as a haplotype (not as single SNPs). Future studies in this field are warranted to gain a better understanding of the interactions between the *FTO* gene polymorphisms and anthropometric measurements.
